# Bilateral Tubal Pregnancy without Known Risk Factor

**DOI:** 10.1155/2017/4356036

**Published:** 2017-10-18

**Authors:** Hyacinthe Zamané, Barnabé Yameogo, Paul Dantola Kain, François Gueswendé Xavier Kaboré, Yobi Alexis Sawadogo, Sibraogo Kiemtoré, Sidbewenné Yacinthe Kaboré, Blandine Bonané Thiéba

**Affiliations:** ^1^Department of Obstetrics and Gynecology, Yalgado Ouedraogo Teaching Hospital, 03 P.O. Box 7022, Ouaga 03, Burkina Faso; ^2^Unit of Training and Research in Health Sciences (UFR/SDS), University Ouaga I Prof. Joseph KI-ZERBO, 03 P.O. Box 7021, Ouaga 03, Burkina Faso; ^3^Unit of Obstetrics and Gynecology, Regional Hospital of Tenkodogo, P.O. Box 56, Tenkodogo, Burkina Faso

## Abstract

Spontaneous bilateral ectopic gestation is very rare. The authors report a case diagnosed and taken care of at Yalgado Ouedraogo Teaching Hospital, Ouagadougou. It was a 30-year-old patient with no known pathological history. She had presented at the obstetric emergencies with a state of hypovolemic shock by haemoperitoneum with digestive disorders, pelvic pain, vaginal bleeding, and a mention of delayed menstruation. The ultrasound coupled with the urinary immunological pregnancy test confirmed the diagnosis of ruptured ectopic pregnancy and a bilateral form was suspected. A laparotomy in emergency confirmed the diagnosis of bilateral ectopic gestation with a right ampullary unruptured pregnancy and a left isthmic ruptured gestation. A bilateral salpingectomy was performed and counseling was made for the use of medical help of procreation in case of future need of pregnancy.

## 1. Introduction

Ectopic pregnancy is an ectopic implantation of the egg outside the uterine cavity. The bilateral form is rare [1, 2]. The study of the few cases described in the literature shows a close correlation between this entity and medical technical of help for procreation [3, 4]. The spontaneous cases are exceptional. The authors report a case of bilateral spontaneous ectopic gestation diagnosed and taken care of in the Department of Obstetrics and Gynecology of Yalgado Ouedraogo Teaching Hospital.

## 2. Case Study

It was a 30-year-old patient who was fourth gravida and third para with no particular pathological antecedent known. She was admitted to the obstetrical emergencies on account of loss of consciousness preceded by vomiting, pelvic pain, and vaginal bleeding which occurred 72 hours earlier in a context of delayed menstruation. At the admission, the blood pressure was 100/40 mmHG; the pulse was at 110 pulses/mn; the heart rate was at 108 beats/mn. To those were added a conjunctival and palmoplantar pallor with cold at extremities of upper and lower limbs. In addition to the state of shock, there were signs of peritoneal irritation with a vaginal bleeding. The culdocentesis has brought out back 5 mls of incoagulable blood. The urinary standard pregnancy test was positive and the rate of hemoglobin was at 6.1 g/dl.

An emergency pelvic scan done has shown an empty uterus but with a thick endometrium, deciduous appearance. The Douglas dead-end was a site of heavy liquid effusion of sufficient abundance. In addition, there was a left lateral uterine mass of 4 cm of diameter with cockade image containing a vesicle with an embryo with no cardiac activity. To the right, there was doubt about a heterogeneous lateral uterine mass with an irregular outline with an ultrasound appearance of blood clots all bathing in a liquid effusion. The diagnosis of a ruptured ectopic pregnancy complicated by shock was made and a bilateral form was suspected.

**Figure 1:**
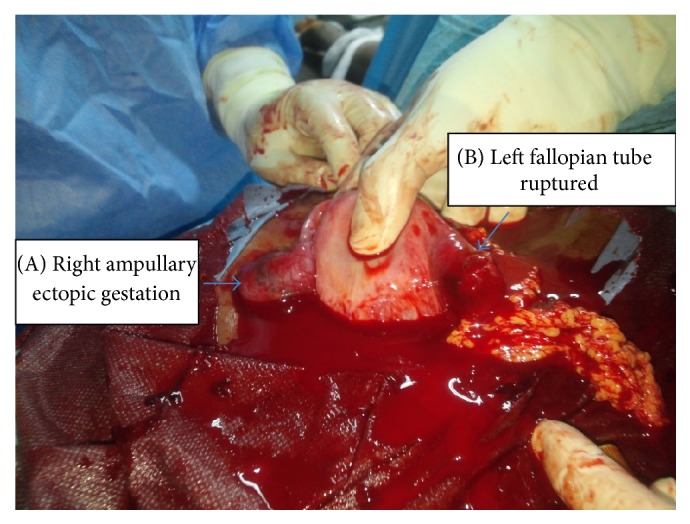
Peroperative view of bilateral ectopic pregnancy: (A) right ampullary unruptured pregnancy; (B) left fallopian tube ruptured with a few scraps around.

The patient had an emergency laparotomy with macromolecular resuscitation, blood products transfusion, and intravenous antibiotic therapy. Peroperative exploration has found haemoperitoneum of great abundance. The right fallopian tube had an ampullary hematosalpinx of 3 cm of long and the left fallopian was an isthmic ruptured of ectopic pregnancy with scraps inside (Figure 1). The diagnosis of bilateral ectopic gestation was made. A bilateral salpingectomy was then performed. Histological analysis has concluded to the same diagnosis. Counseling has been done to the couple on the necessity to resort to medical help of procreation in case of future need of child.

## 3. Comments

Spontaneous bilateral ectopic gestation without assisted reproduction or IVF/ICSI is very rare with an incidence of 1 for 725 to 1 for 1580 ectopic pregnancies [5]. Classically, it is recognized that the risk factors of ectopic gestation are multiples [6]. Assisted reproduction is the one main risk factor of bilateral ectopic [3, 7]. Approximately half of all women with ectopic pregnancies do not have any known risk factors. Indeed, in our case, no risk factor was found. Other authors as Hoffmann et al. [8] as well as Barnhart et al. [9] have found the same findings.

The bilateral ectopic gestation is a delayed diagnosis and it is made at the complications stage [4]. The diagnosis of bilaterally, difficult before surgery, is generally made during operation [8, 10] and hence the need to always check the other fallopian tubes during the intervention for ectopic gestation.

*β*-hCG detection has no contribution [5, 11]. In our case, the diagnosis was made at the stage of rupture stage with hypovolemic shock.

Ultrasound scan has shown two lateral uterine masses: one was highly suspect of an ectopic pregnancy and the other doubtful aspect with a haemoperitoneum appearance of great abundance. The diagnosis of bilateral ectopic gestation was made in peroperative with a visualization of a left isthmic ruptured ectopic gestation and a right ampullary mass corresponding to an unruptured ectopic pregnancy.

The management of ectopic gestation is variable [2]. It can be medical, surgical with conservation, or not depending on the localization of pregnancy, the status of fallopian tube during the diagnosis, and the need of future pregnancy [8]. Usually, a surgical act is needed; the medical treatment is for particular circumstances [12, 13]. Salpingotomy is often preferred to salpingectomy in order to conserve the fertility although there is little evidence which supports that management [14]. The conservative treatment may lead to recurrence of ectopic gestation and the continuing of trophoblastic disease if a molar pregnancy was associated [8]. In our case, a bilateral salpingectomy was achieved. That attitude reported by other authors [10, 15] constitutes a drama on fertility side mainly in our setting where medical help for procreation remains restricted.

## 4. Conclusion

Bilateral ectopic pregnancy is very rare. Efforts should be done to make the diagnosis earlier in order to improve the prognosis of future fertility.
